# The Impact of the High-Energy Grinding of Wood Ash on Its Pozzolanic Activity

**DOI:** 10.3390/ma18133100

**Published:** 2025-06-30

**Authors:** Ece Ezgi Teker-Ercan, Rafał Panek, Maciej Szeląg, Andrzej Cwirzen, Karin Habermehl-Cwirzen

**Affiliations:** 1 Building Materials Group, Department of Civil, Environmental and Natural Resources Engineering, Luleå University of Technology, 97187 Luleå, Sweden; andrzej.cwirzen@ltu.se (A.C.); karin.habermehl-cwirzen@ltu.se (K.H.-C.); 2 Faculty of Civil Engineering and Architecture, Lublin University of Technology, 40 Nadbystrzycka Str., 20-618 Lublin, Poland; r.panek@pollub.pl (R.P.); maciej.szelag@pollub.pl (M.S.); 3 Faculty of Science and Technology, University of the Faroe Islands, FO-100 Torshavn, Faroe Islands

**Keywords:** wood ash, mechanochemical activation (MCA), ball mill, pozzolanic property, SAI, R3 test, Frattini test, TGA/DTG

## Abstract

Wood ash is a promising supplementary cementitious material (SCM) due to its inherent pozzolanic properties. Intensive grinding has been shown to enhance this aspect and reduce the negative effects of variability in the chemical composition. This study investigated the influence of grinding through ball milling on the pozzolanic properties of wood ash. Four different types of wood ash were studied, each subjected to grinding durations of 10 and 20 min. Coal fly ash was used as a reference material. The pozzolanic activity of raw and ground wood ashes was evaluated using the strength activity index (SAI), the Frattini test, the R3 test, thermogravimetric analysis (TGA/DTG), X-ray diffraction (XRD) analysis, and scanning electron microscopy with energy-dispersive spectroscopy (SEM/EDS). The results indicated that both 10 min and 20 min grinding durations enhanced the reactivity and compressive strength. However, the 10 min grinding duration showed better overall performance than 20 min grinding, likely due to reduced agglomeration and more effective particle refinement. For calcium-rich wood ashes, the reactivity was linked to the hydraulic properties rather than the pozzolanic properties.

## 1. Introduction

Supplementary cementitious materials (SCMs) are fine powders that are used to partially replace Portland cement [1,2]. They react with calcium hydroxide (Ca(OH)_2_) formed during the hydration of Portland cement via a pozzolanic reaction [3,4]. This results in the formation of an additional calcium silicate hydrate (C-S-H) gel [2,5,6]. Interest in pozzolans is increasing because they reduce the overall environmental impact and cost when used as a partial replacement for Portland cement. Moreover, pozzolans might increase the strength and enhance the durability of concrete [6]. Several SCMs, such as fly ash, slag, silica fume, biomass ashes, calcinated clays, etc., have been tested for their pozzolanic properties [1]. There are many different test methods for the assessment of pozzolanic activity, and they can be categorized as direct or indirect. Direct methods include the Frattini test, the Chapelle test, the saturated lime method, and analytical methods such as X-ray diffraction (XRD) or thermogravimetric analysis (TGA). These methods monitor the presence of Ca(OH)_2_ and the decrease in the amount of calcium ions over time due to the pozzolanic reaction [6,7]. Indirect methods, which include the strength activity index (SAI), electrical conductivity test, and rapid, relevant, and reliable (R3) test, etc., measure some physical properties, such as the compressive strength, electrical conductivity, or heat evolution, by calorimetry [6,8].

The widespread use of fly ash as an SCM has previously been due to its availability, its ability to improve concrete’s properties, and its cost-effectiveness [8]. However, despite its past availability, fly ash use is now declining globally due to the gradual shutdown of coal-based thermal power plants. Wood ash, derived from burning wood and wood products, is considered an alternative to coal fly ash [9]. Wood ash contains both organic and inorganic residues, constituting about 6–10% of the weight of the wood burned [10]. Globally, approximately 4.6 billion tons of wood biomass are produced annually, with 60% used for energy production, 20% for industrial purposes, and the remaining 20% disposed of in landfills [11].

The quality control of wood ash is more complicated than that of coal fly ash due to variations in organic and inorganic content. This content is influenced by the tree species, geographical location, combustion technology, combustion temperature, and collection and storage methods [12,13,14,15,16]. Due to these factors, the composition of wood ash can differ significantly, making it challenging to ensure consistent quality. However, studies indicate that wood ash might enhance the strength and durability of concrete [17,18,19]. In addition, beneficial effects have been observed when wood ash is pre-treated with methods like sieving, grinding, calcination, and washing. These pre-treatments can help to standardize the material and improve its performance in construction material applications [12,20,21,22].

Some researchers have also reported on the pozzolanic properties of wood ash. Elinwa and Mahmood [23] stated that the tested wood ash exhibited pozzolanic properties due to its high pozzolanic oxide content of 73.55%. Rajamma et al. [18] reported lower pozzolanic oxide content of 53.2% but still confirmed the pozzolanic activity of wood ash using the Frattini test. Kostanić Jurić et al. [24] tested various wood ashes and observed pozzolanic and hydraulic properties in some longer-aged samples via the SAI and Frattini tests. Conversely, Garcia and Sousa-Coutinho [17] used the Frattini test and did not detect pozzolanic properties in any of the tested wood ashes. Demis et al. [25] attributed the absence of pozzolanic activity in wood ash to its low SiO_2_ content. Berra et al. [12] also did not observe pozzolanic activity in their study.

Mechanochemical activation (MCA), achieved through grinding with a ball mill, has been widely utilized to enhance the reactivity of various supplementary cementitious materials (SCMs), such as fly ash and clays [26,27,28]. This process decreases the particle size, increases the specific surface area, and induces structural disorder and amorphization, all of which enhance the dissolution of reactive species and promote hydration reactions [29,30]. Mucsi [31] emphasized the importance of crystal defects and structural decomposition, while Kumar and Kumar [32] highlighted network depolymerization and increased surface reactivity in geopolymer systems.

In recent years, MCA has increasingly been used for biomass ashes to improve their reactivity. Several studies have reported that grinding significantly reduces the particle size and increases the surface area, thus enhancing hydration and strength development. For example, Kaminskas and Cesnauskas [33] found that the vibratory milling of biomass fly ash from wood chips reduced 70 wt.% of particles to below 63 µm and enhanced C–S–H and portlandite formation, allowing for up to 15 wt.% cement replacement without compromising the strength. Similar findings were observed by Rosales et al. [21] for biomass bottom ash and by Xu et al. [34] for rice husk ash, with optimal milling times improving the reactivity and reducing the porosity. Pantić et al. [35] noted that grinding increased the nucleation sites, refined the pores, and enhanced filler packing, all of which contributed to better early hydration and durability. Ohenoja et al. [36] also reported improved self-hardening behavior in peat biomass fly ash through the refinement of the particle size distribution.

In addition to physical refinement, the chemical composition of the ash affects the MCA efficiency. Šupić et al. [37] showed that high-silica ashes responded effectively to grinding alone, while low-silica ashes required chemical activation. de Moraes et al. [38] reported that prolonged grinding enhanced the reactivity of initially inert sugarcane bagasse ash by increasing its amorphous content and surface area, resulting in higher long-term strength. Ke et al. [30] showed that MCA could significantly improve the carbonation potential of timber biomass ash by disrupting polymerized Si-O-Si bonds and creating active oxygen sites. This process enabled partial mineral carbonation even at ambient temperature and pressure within 1–2 h. CO_2_ uptake was increased by up to 100%, even for ashes with low alkali content. These findings suggest that MCA can also serve as a pre-treatment method to enhance calcium/magnesium silicate-rich industrial residues in carbon capture applications.

While numerous studies have examined the mechanochemical activation of various biomass ashes, including some wood ashes, few have investigated how different grinding durations impact the pozzolanic activity of various wood ash types using a combination of direct and indirect testing methods.

This paper explores the impacts of grinding using a ball mill on the pozzolanic properties of wood ash in comparison to fly ash. Four different wood ashes were examined, each undergoing two different grinding durations: 10 or 20 min. The enhancement of the pozzolanic properties was assessed using the strength activity index (SAI), Frattini test, R3 test, X-ray diffraction (XRD) analysis, thermogravimetric analysis (TGA/DTG), and scanning electron microscopy with energy-dispersive spectroscopy (SEM/EDS). The findings from these analyses provide insights into how ball milling affects the reactivity of wood ash particles, which is essential in evaluating their potential as a supplementary cementitious material.

## 2. Materials and Methods

### 2.1. Materials

Four different wood fly ashes (WA1, WA2, WA3, and WA4), which were byproducts of timber waste combustion, were used in this project. WA1, WA2, and WA3 were obtained from Stenvalls Trä AB (Piteå, Sweden) and were collected at different times. WA4 was obtained from Holmen AB (Skellefteå, Sweden). The production parameters for each wood ash, including the biofuel composition, combustion technology, and combustion temperature, are summarized in Table 1. The wood ashes were dried in an oven at 65 ± 0.5 °C for 24 h and sieved with a 500 µm sieve before grinding [20]. They were then ground using a planetary ball mill at a rotation speed of 500 rpm, with a ball-to-powder ratio of 5. The grinding process was conducted for two different durations of 10 and 20 min [39]. The equipment used was a Retsch PM100 planetary ball mill (Retsch GmbH, Haan, Germany), with a stainless-steel jar having a 500 mL capacity and 30 stainless-steel balls with a diameter of 10 mm. The particle size distribution (PSD) of the wood ashes, fly ash, and cement was determined with the laser diffraction method using a Mastersizer 3000 analyzer by Malvern Instruments (Worcestershire, UK). Isopropanol was used as the dispersing medium, with an ultrasonic probe employed to disaggregate larger grain clusters. The analysis covered grains ranging from 0.1 µm to 2000 µm in equivalent diameter. The chemical compositions of WA1, WA2, WA3, WA4, FA, and CEM I, the sum of pozzolanic oxides (SiO_2_ + Al_2_O_3_ + Fe_2_O_3_), and the ratios of CaO/SiO_2_ and (CaO + MgO)/SiO_2_ are provided in Table 2. The missing percentages in the sum of oxides and LOI of the wood ash samples may be attributed to the high sulfur content that was released during combustion [39].

Portland cement (CEM I 42.5 N) and coal fly ash (Type F) were obtained from Cementa (Skövde, Sweden) and Thomas Cement (Bremen, Germany), respectively. Fly ash (FA) was used as a reference for the SAI, Frattini test, R^3^ test, TGA/DTG, XRD, and SEM/EDS analyses.

### 2.2. Testing Methods

#### 2.2.1. Strength Activity Index (SAI)

The control mix with 100 wt.% Portland cement and the test mix with 20 wt.% wood ash or fly ash were prepared according to ASTM C311 [40]. Graded standard sand (0–4 mm) was used in the mortar mixes with an s/b ratio of 2.75, and the w/b ratios were 0.484 for the control sample and 0.494 for the test samples to maintain the required flow, according to ASTM C311 [40]. Mixing was performed using a 3 L Hobart mixer, where dry materials were blended for 3 min, followed by the addition of water and mixing for an additional 2 min. The casting was carried out in cube molds with dimensions of 50 × 50 × 50 mm^3^. After 24 h, all samples were demolded and submerged in a water bath until the days of testing.

The strength activity index (SAI) was assessed by an average of three compressive strength measurements on mortars at the age of 7 and 28 days and calculated via Equation (1), which is specified in ASTM C618 [41]:

(1) $$SAI\left(\%\right)=\frac{A}{B}\times 100$$

where A is the compressive strength value of the 20 wt.% wood ash or fly ash containing mortar cubes, and B is the compressive strength of the 100 wt.% OPC containing control mortar cubes.

#### 2.2.2. Frattini Test

The Frattini test was used to evaluate the pozzolanic reactivity of wood ash by EN 196-5 [42]. To conduct the test, a mixture of 80 wt.% Portland cement and 20 wt.% wood ash or fly ash was prepared and compared to a control sample consisting of 100 wt.% Portland cement (PC). All samples were mixed with 100 mL of distilled water that had been initially boiled and then stored in sealed containers in an oven at 40 °C. After 8 days, the samples were vacuum-filtered through filter paper, and 50 mL of the filtrate was titrated with 0.1 M hydrochloric acid (HCl), using methyl orange as the indicator. Then, [Ca^2+^] ions were titrated with a 0.3 M EDTA solution using Patton and Reeder’s indicator. The concentrations of hydroxyls and [Ca^2+^] ions were then plotted, illustrating the relationship between the CaO concentration and [OH^−^] ion concentration [6,26,43]. The test was conducted again on the 15th and 28th days for the samples that did not exhibit pozzolanic properties on the 8th and 15th days, respectively. As stated in the standard [42], it is not necessary to repeat the test at later ages for test samples that show pozzolanic properties on the 8th day. Since EN 196-5 provides lime solubility data for [OH^−^] concentrations between 35 mmol/L and 90 mmol/L, the lime solubility curve was plotted according to the theoretical [CaO] concentration calculated via Equation (2) for [OH^−^] concentrations between 40 mmol/L and 110 mmol/L [6]:

(2) $$Max[CaO]=\frac{350}{\left[OH\right]-15}$$

#### 2.2.3. R3 Test

The heat of hydration of the pastes was measured according to ASTM C1897–20 [44] using an 8-channel TAM Air isothermal calorimeter. The potassium solution was prepared by dissolving 20 g of potassium sulfate powder and 4 g of potassium hydroxide pellets in 1 L of distilled water. The mixture was maintained in an airtight container at 40 ± 2 °C [8]. All dry materials, which consisted of wood ashes, fly ash, calcium hydroxide, and calcium carbonate, were weighed and kept at 40 °C overnight. The mix proportions of the SCM, calcium hydroxide (Ca(OH)_2_), and calcium carbonate (CaCO_3_) were 1:3:0.5, and the liquid-to-solid ratio was 1.2:1 by mass, as specified in ASTM C1897–20 [44]. The dry and wet materials were mixed for 2 min in a small-volume vacuum mixer, namely an Ecovac device (Bredent/Senden, Germany), at a mixing speed of 600 rpm. Then, 15 g of freshly mixed paste was placed into a glass ampoule, which was sealed quickly. The measurements were carried out for 7 days at a 40 ± 0.5 °C base temperature. The cumulative heat release of test samples was calculated using Equation (3), ranging from 75 min to 168 h:

(3) $$H_{SCM}=\frac{H}{{(m}_{p}\times 1.101)}$$

where *H* is the cumulative heat release, *m_p_* is the mass of the paste, and 0.101 is the mass fraction of the SCM in the paste.

#### 2.2.4. X-Ray Diffraction (XRD) Analysis

The X-ray diffraction (XRD) analysis was performed on powdered samples and 7- and 28-day-old pastes by using the Empyrean platform from PANalytical with a PIXcel 3D detector (Malvern, UK), at operating conditions of 45 kV and 40 mA, using Cu-K radiation with a wavelength of 1.54 Å. The step size was 0.02, with a 2θ angle range of 5° to 70° [45]. The Profex software (v.5.2.8) and the Crystallography Open Database (COD) were used to determine the phase composition. Using the Quick Peaks Gadget in OriginPro 2024, a semiquantitative analysis was performed to calculate the approximate portlandite peak areas for 7- and 28-day-old pastes [26].

#### 2.2.5. Thermogravimetric Analysis (TGA/DTG)

Thermogravimetric analysis (TGA) was performed on the same paste samples used for the XRD, SEM, and EDS analyses. The 28-day-old paste samples were crushed and ground using a mortar and pestle to achieve a particle size of ≤1 mm. Then, a solvent exchange procedure was performed, following the method described by Scrivener et al. (2016) [46]. Approximately 5 g of the ground paste was immersed in 50 mL of isopropanol and left to rest for 10 min. The suspension was filtered using a vacuum filter, and the sample was washed with 10 mL of diethyl ether to remove residual isopropanol, followed by a second filtration step. Finally, the samples were oven-dried at 40 °C for 8 min. TGA was conducted to measure the weight loss while heating the samples from 20 to 1000 °C at a rate of 20 °C/min using a NETZSCH (Selb, Germany) STA 449 F3 Jupiter instrument [46]. Approximately 50 mg of powdered sample was placed in alumina crucibles, and Ar was used as the purging gas at a flow rate of 50 mL/min. The horizontal step was used to identify the loss of bound water and the decomposition of ettringite and calcite, while the tangential method was used to determine the decomposition of portlandite [46,47]. The equations and temperature ranges used for the quantification of bound water (H), portlandite (CH), estimated ettringite (Ett), and calcite (Cc) from the TGA data are summarized in Table 3 [46,48]. The measured phase content was normalized to the anhydrous binder weight based on the residual mass at 550 °C. The mass loss observed in the 50–120 °C range was used to estimate the ettringite content based on stoichiometric calculations commonly applied in previous studies [49,50]. It is important to note that, while this range primarily corresponds to ettringite, there may also be contributions from loosely bound water in C–S–H or AFm phases, if present [49].

Table 4 summarizes the types of samples, mix designs, and methods used in each test described above.

#### 2.2.6. Microstructural Investigation

The microstructures of the hardened paste samples and morphologies of the wood ashes were examined using a scanning electron microscope (SEM), JSM-IT100 (JEOL Ltd., Tokyo, Japan), connected with an energy-dispersive spectrometer, BRUKER (JEOL Nordic AB, Sollentuna, Sweden), and the ESPRIT software (v.2.1).

The 7- and 28-day-old paste samples were kept in isopropanol for 48 h to stop ongoing reactions. Then, the samples were stored in a desiccator for 48 h [51]. In the next step, the samples were impregnated with a Struers EpoFix low-viscosity epoxy resin under a vacuum. After the resin had hardened, the samples were polished with grinding plates covered with a diamond spray having particle sizes of 9, 3, and 1 µm. A paraffin-based lubricant was used, and the samples were cleaned in an ultrasonic bath filled with isopropanol [52]. The SEM analysis was performed using a backscattered electron detector (BSE) in low-vacuum mode. The accelerating voltage was 15.0 kV at 500× magnification. EDS spot analysis was also performed at a magnification of 1000× by choosing 100 points manually based on the grey-level histogram that corresponded to C-S-H [52]. The powder samples were dispersed onto conductive tape to analyze the morphologies of the wood ashes.

## 3. Results and Discussion

### 3.1. Wood Ash Characterization

**Figure 1:**
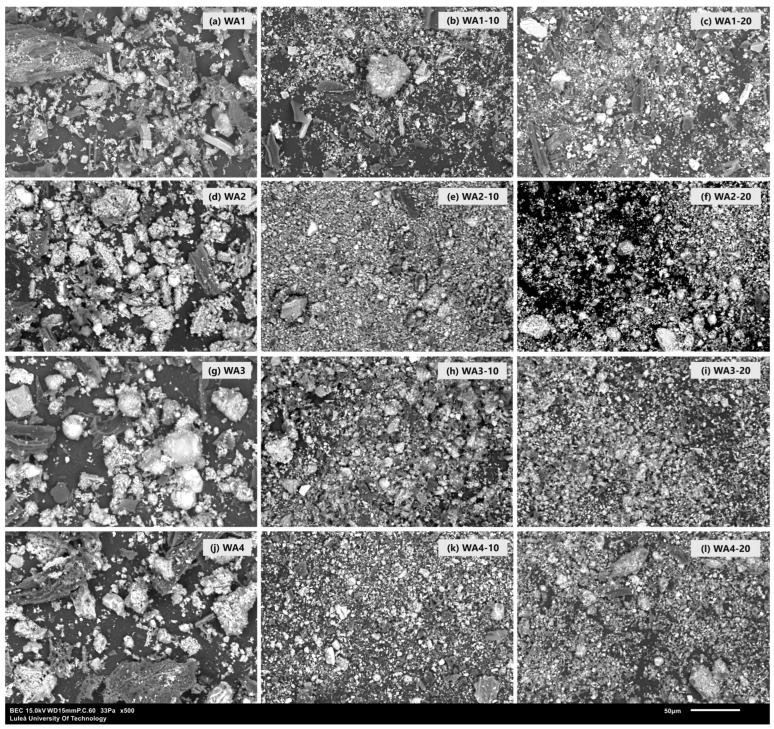
SEM images of wood ash powders before and after grinding for 10 and 20 min, captured at 500× magnification. The grinding process significantly altered the particle size and morphology, resulting in finer, more uniform particles with reduced angularity and porosity.

**Figure 2:**
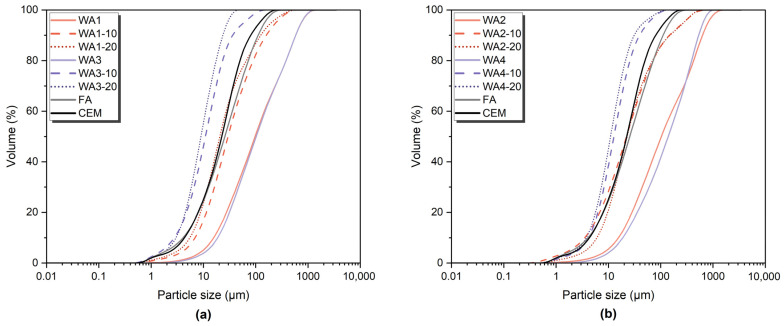
Cumulative particle size distribution curves of unground and ground wood ashes—(**a**) WA1 and WA3 and (**b**) WA2 and WA4—compared to fly ash (FA) and Portland cement (CEM I).

The SEM images of unground and ground WA1, WA2, WA3, and WA4 are given in Figure 1. All studied wood ashes had a similar morphology, with large and irregularly shaped particles. Grinding with a ball mill significantly reduced the particle size, resulting in more uniform and refined shapes. These morphological changes are clear in Figure 1, where the ground samples (10 and 20 min) show smoother and smaller particles compared to the unground wood ashes, demonstrating the effectiveness of the grinding process in enhancing the physical fineness. The particle size distributions of untreated wood ashes (WA1, WA2, WA3, and WA4), wood ashes ground for 10 min (WA1-10, WA2-10, WA3-10, and WA4-10), and wood ashes ground for 20 min (WA1-20, WA2-20, WA3-20, and WA4-20), as well as FA and CEM I, are given in Figure 2. The corresponding values of the 10th (D10), 50th (D50), and 90th (D90) percentile particle sizes and specific surface areas (SSAs) are given in Table 5.

Before grinding, the wood ash particle sizes ranged from 97.2 µm to 139 µm, whereas fly ash and cement exhibited smaller D50 values of 25.6 µm and 22.4 µm, respectively. After 10 min of grinding, the particle sizes of wood ash decreased significantly, with D50 values of 29.5 µm for WA1, 22.2 µm for WA2, 10.3 µm for WA3, and 12.7 µm for WA4. However, the following 20 min of grinding resulted in a further decrease for WA1, WA3, and WA4 to 20.6 µm, 8.98 µm, and 11 µm, respectively. WA2 showed a slight increase to 22.8 µm. This behavior is likely caused by particle agglomeration. Additionally, the D10 values, which represented the finest fractions of the particles, significantly decreased with grinding, especially in WA3 and WA4. As the particle size decreased, the specific surface areas of the wood ash samples showed a significant increase with longer grinding. The specific surface areas of the untreated wood ash samples ranged from 131.9 to 186.6 m^2^/kg. After 10 min of grinding, these values increased considerably, with that of WA3-20 reaching as high as 1048 m^2^/kg. However, after 20 min of grinding, WA2 exhibited a slight reduction in specific surface area compared to the 10 min values, consistent with the trend in the particle size. In contrast, WA1, WA3, and WA4 showed a continuous decrease in particle size and a corresponding increase in specific surface area.

Although grinding generally reduces the particle size, excessive grinding beyond a critical intermediate time might negatively impact particle size reduction [39,53,54,55]. Agglomeration widens the particle size distribution, with most changes occurring within the first hour of grinding. Kumar and Kumar [32] reported that a significant reduction in particle size happens within the initial 10 min of the fly ash grinding process, with a decreasing impact afterward. Prolonged grinding processes might lead to more irregular particle morphologies than shorter durations [54,55]. Furthermore, smaller particles in the grinding media can gradually cover the grinding balls, diminishing the grinding process’s efficiency over time [56]. The wear and tear of the grinding media, which might lead to contamination, also significantly influences the grinding kinetics, reducing the overall grinding efficiency [57].

According to EN 450-1 [58], the sum of SiO_2_, Al_2_O_3_, and Fe_2_O_3_ should not be less than 70%. However, none of the studied wood ashes met this requirement (Table 1). WA1 had the highest and WA4 had the lowest pozzolanic oxide content, which were 43.14% and 1.29%, respectively.

Wood ash containing a high amount of CaO (>20%) might show hydraulic properties [59]. The hydraulic activity is primarily determined by the presence of SiO_2_ and CaO. According to EN 197-1 [60], the ratio of CaO/SiO_2_ should exceed 2. WA2 and WA4 met the requirements of hydraulic materials specified in EN 197-1 [60]. Many researchers [12,18,49] have investigated the hydraulic index (K_3_), which is calculated as (CaO + MgO + Al_2_O_3_)/SiO_2_ and should be greater than 1 to indicate good hydraulic properties [12,61].

The LOI values of the wood ashes ranged from 29.7% to 56.7%, which are relatively high compared to those reported in the literature (0.47–74.31% [62,63]). Such high LOI values are usually associated with unburnt carbon but can also result from pre-combustion organics or the formation of carbonates like CaCO_3_ and K_2_Ca(CO_3_)_2_ during combustion [64,65]. These values are influenced not only by the combustion technology, temperature, and biofuel composition but also by the collection method and period. Inconsistent furnace operation or extended retention times can cause the buildup of volatile and partially reacted phases [66].

**Figure 3:**
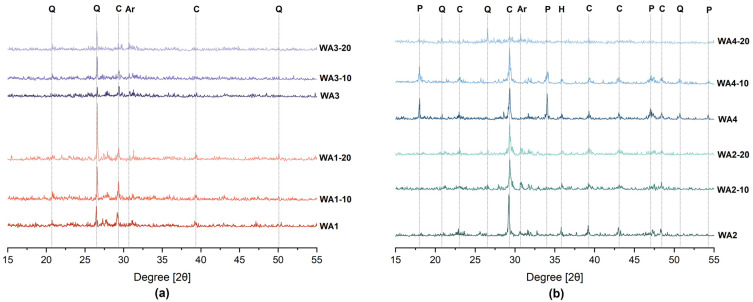
XRD patterns of unground and ground wood ashes: (**a**) WA1 and WA3; (**b**) WA2 and WA4 (P = Portlandite, C = Calcite, Q = Quartz, Ar = Arcanite, H = Hematite). Line colors represent different ash types and grinding durations: orange shades correspond to WA1, purple to WA3, green to WA2, and blue to WA4. For each ash type, lighter shades indicate longer grinding durations (unground, 10 min, and 20 min, from darkest to lightest).

The XRD patterns of the unground and ground wood ashes are given in Figure 3. Due to the similar peaks observed, WA1, WA1-10, and WA1-20, as well as WA3, WA3-10, and WA3-20, are displayed together in Figure 3a. Similarly, WA2, WA2-10, and WA2-20, as well as WA4, WA4-10, and WA4-20, are displayed together in Figure 3b.

The main crystalline phase of WA1, WA1-10, and WA1-20 was quartz (Q), as was the case for WA3-10, WA3-20, and WA4-20. The main crystalline peak of WA3 was calcite (C). The intensity of the quartz peak for WA1 and WA3 increased with longer grinding times. One possible explanation for this phenomenon is the hardness of quartz, which allows it to remain mostly unaffected by the grinding process, while quartz itself exhibits slower amorphization compared to other minerals [67]. Moreover, its presence can accelerate the amorphization of less resistant minerals by acting as a grinding medium during prolonged grinding [68]. As a result, quartz may become the predominant crystalline phase following the amorphization of these minerals [69]. Calcite, with varying intensities, was present in both ground and unground samples of WA1 and WA3. Additionally, arcanite (Ar) was found in both the unground and ground samples of WA3, WA3-10, and WA3-20.

Calcite was the dominant crystalline peak in WA2, WA2-10, WA2-20, WA4, and WA4-10. The intensity of the calcite peak decreased after grinding, with WA2-10 having a lower intensity than WA2-20. Furthermore, portlandite and quartz were observed in all these samples. The calcite peak intensity of WA4 increased after 10 min of grinding, but the calcite peak disappeared after 20 min. Hematite was observed in all ashes except WA4-20 but at low intensities. High grinding energy can cause changes in the surfaces of minerals and carbonates in the material, reducing their crystallinity and weakening the binding energy of the surface Al-O or Si-O bonds [30]. A decrease in peak intensity was observed in all phases in WA2 and WA4 after grinding. Kato et al. [55] observed amorphization with increasing grinding durations in all crystalline phases. They also explained three main stages in the mechanochemical activation of fly ash. The first stage involves grinding large ash particles using mechanical energy. In the second stage, the mechanical energy causes the crystals to become amorphous as the coarse particles become smaller and reach the grinding limit. In the final stage, the mechanical energy causes the formation of agglomerates, which can then be ground again. It is important to optimize the effects of the grinding process combined with activation.

### 3.2. Strength Activity Index (SAI)

**Figure 4:**
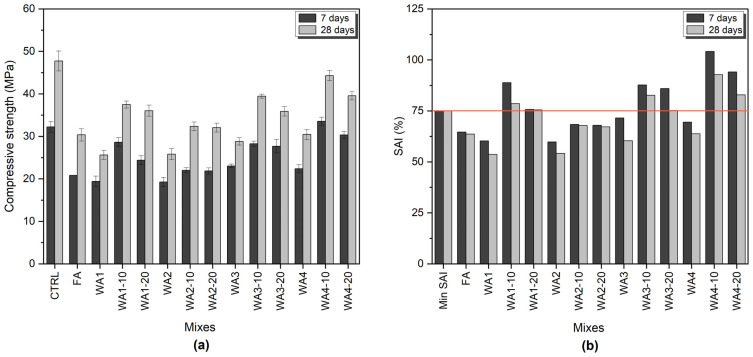
(**a**) Compressive strength of mortar samples containing 20 wt.% fly ash or wood ash (unground and ground) and a control sample with 100 wt.% cement; (**b**) strength activity index (SAI) at 7 and 28 days. The orange line indicates the minimum SAI requirement (75%) according to ASTM C618 [41] for a material to be classified as pozzolanic.

The average compressive strength of the mortars and the calculated SAI values are given in Figure 4.

A one-way ANOVA was performed using OriginPro 2025 (v.10.2.0.188) and showed statistically significant differences in compressive strength between the mixes at both curing ages (7 days: *p* < 0.0001, 28 days: *p* < 0.0001). According to the Tukey test (*p* < 0.05), WA1 and WA2 had significantly lower compressive strength than CTRL, while WA4-10 reached a statistically similar value to CTRL. Samples containing ground wood ashes also exhibited significantly higher compressive strength than those with unground wood ashes. These results clearly show the effectiveness of grinding in enhancing the mechanical performance of systems containing wood ash.

The control mortar (CTRL), containing 100 wt.% of OPC, reached 32.22 MPa and 47.74 MPa after 7 and 28 days, respectively. Both 10 and 20 min grinding durations improved the compressive strength of WA-containing mortars, and 10 min grinding increased the compressive strength compared to 20 min grinding for all mortars. Grinding wood ash for 10 min increased the compressive strength of the mortars by 14.3% to 49.7% at 7 days and by 25.2% to 46.2% at 28 days compared to using unground wood ash. On the other hand, grinding for 20 min resulted in an increase in compressive strength of 13.6% to 35.4% at 7 days and of 24% to 40.6% at 28 days compared to unground wood ash. Moreover, mortars containing 20 wt.% of WA4-10 exhibited improved compressive strength by 4% after 7 days, but an 11% reduction was observed after 28 days compared with CTRL.

According to ASTM C618 [41], a material can be classified as having pozzolanic reactivity if the SAI is higher than 75% for both 7 and 28 days. Ground WAs, except WA2-10 and WA2-20, exceeded the limit for SAI and exhibited pozzolanic properties, as specified in ASTM C618 [41], at both 7 and 28 days. However, unground WAs (WA1, WA2, WA3, and WA4) and FA did not exceed 75%. A reduction was observed for the SAIs of all samples from 7 to 28 days. Grinding positively impacted all ashes in terms of compressive strength improvement, but only WA1, WA3, and WA4 showed pozzolanic activity according to the SAI. Donatello et al. [28] attributed the increase in the compressive strength of the grinding process to the following possibilities: reduced water in the mortar, an increased amorphous silica surface area of the ground ash resulting in higher pozzolanic reactivity, ground fine particles acting as fillers, or a combination of all of these.

The SAI is a commonly used indirect test parameter for the evaluation of the pozzolanic properties of a material. Many researchers [17,24,48] have reported that the SAIs of wood ash-containing samples exceed the SAI limit, and washing treatment also increases the SAI [48]. However, the test results may be misleading due to nucleation sites and particle packing effects [48]. Donatello et al. [28] suggested increasing the required SAI limit or increasing the cement replacement level from 20 wt.% to 25 wt.% or 30 wt.% in the ASTM C618 [41] requirements to prevent these misleading results.

Some SCMs might show pozzolanic reactivity at later ages. Increasing the curing time may be a possibility for slow-reacting materials [28]. Moreover, it is difficult to distinguish other hydrate phases’ contributions to the compressive strength from those resulting from pozzolanic activity [48]. Low SAI values were obtained for all unground WAs and ground WA2-containing samples. The reason might be that the formation of hydrates does not significantly contribute to compressive strength development, and also they do not act as fine fillers to support the hydration process [49].

The hydraulic indices of WA2 and WA4 were greater than 2. The increase in the compressive strength of the WA4-containing mortars might be attributed to the hydraulic properties of WA4 [12]. This is also supported by the CaO/SiO_2_ ratio and K_3_ value of WA4, which were 38.67 and 42.03, respectively.

### 3.3. Frattini Test

**Figure 5:**
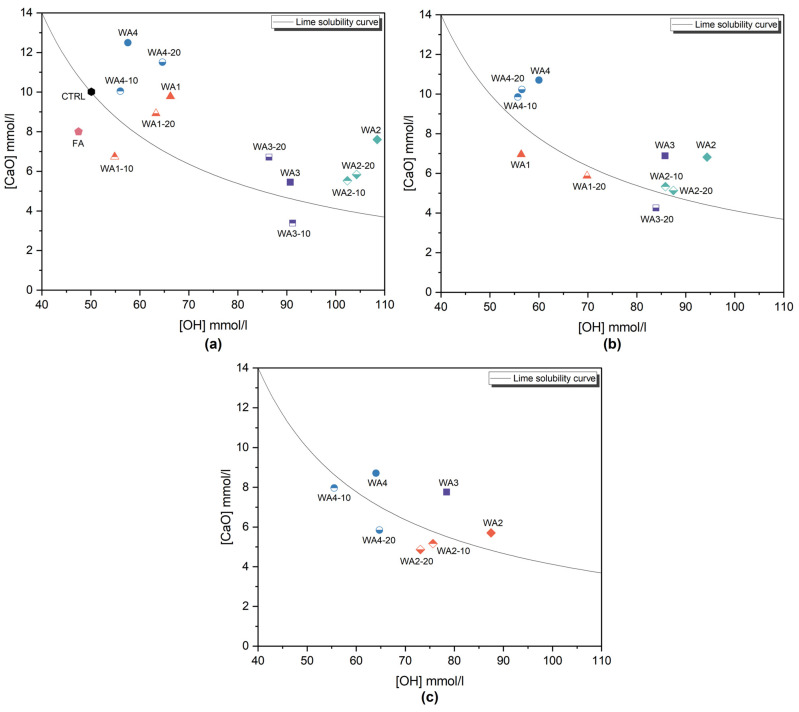
Frattini test results for pastes containing 20 wt.% of wood ash (WA1, WA2, WA3, WA4), ground wood ash (WA1-10, WA1-20, WA2-10, WA2-20, WA3-10, WA3-20, WA4-10, WA4-20) and fly ash (FA) and a control sample of 100 wt.% Portland cement (CTRL) (**a**) after 8 days, (**b**) after 15 days, and (**c**) after 28 days.

The Frattini test results at 8, 15, and 28 days for OPC, FA, and unground and ground WAs are presented as the average of two tested samples for each mixture in Figure 5. Based on EN 196-5 [42], materials with test results below the lime solubility curve are considered to demonstrate pozzolanic reactivity, whereas results above the curve indicate non-pozzolanic properties. The control sample (CTRL), which consisted of 100 wt.% Portland cement, was located on the lime solubility curve.

On the 8th day (Figure 5a), FA, WA1-10, and WA3-10 were located below the lime solubility curve and showed pozzolanic properties. Additionally, on the 15th day (Figure 5b), WA1, WA1-20, and WA3-20 were also located below the lime solubility curve, demonstrating pozzolanic properties and slow reactivity [8].

Although the test days specified in the EN 196-5 standard were 8 and 15, the test was repeated on the 28th day for samples that did not show pozzolanic properties on the 15th day. On the 28th day (Figure 5c), WA2-10, WA2-20, WA4-10, and WA4-20 were also located below the lime solubility curve, indicating slow reactivity development.

The results showed that, except for WA1, all unground WAs were saturated with portlandite for 28 days and did not show enough portlandite consumption to demonstrate pozzolanic characteristics. However, both 10 and 20 min grinding durations enhanced the pozzolanic reactivity of the WAs.

CaO reduction was also calculated via Equation (2) and the values are given in Table 6. Negative values were normalized to 0% equivalent CaO reduction, as Donatello et al. [6] suggested. However, negative values were obtained to show the CaO consumption trend over time. The greatest CaO reduction was observed for WA3-10, which was 26.3%, followed by FA (25.7%) and WA1-10 (23.3%), after 8 days. After 15 days, the CaO removal of WA1, WA1-20, and WA3-20 also increased to 17.8%, 8%, and 16.3%, respectively.

On the other hand, the limited reactivity of both unground and ground WA2 and WA4 may be attributed to their low SiO_2_ content (6.63% and 0.75%, respectively) and high CaO content (22% and 29%, respectively), compared to WA1 and WA3 [8]. Their chemical compositions suggest that the reactivity in WA2 and WA4 is more likely governed by hydraulic behavior rather than pozzolanic reactions.

Some limitations of the Frattini test when applied to high-CaO ashes should be considered, as discussed by Sigvardsen et al. [49]. Wood ashes containing more than 20% CaO are classified as having high calcium mineral additions, which might increase the excess Ca^2+^ concentration, thus affecting CaO reduction. Kostanić Jurić et al. [24] also stated that wood ash containing a small amount of pozzolanic oxides exhibited both pozzolanic and hydraulic properties after 37 days, beyond the testing durations considered in EN 196-5 [42]. These findings suggest that the Frattini test may not fully detect the full reactivity potential of wood ash, especially in samples exhibiting slow or complex hydration mechanisms.

### 3.4. R3 Test—Isothermal Calorimetry

R3, also known as the rapid, relevant, and reliable method, is a recently developed approach to evaluating the reactivity of SCMs through heat of hydration measurements in an isothermal calorimeter. This test was initially designed for blends of calcined clays and limestone, but it is now being used for other SCMs and blends as well [70,71].

**Figure 6:**
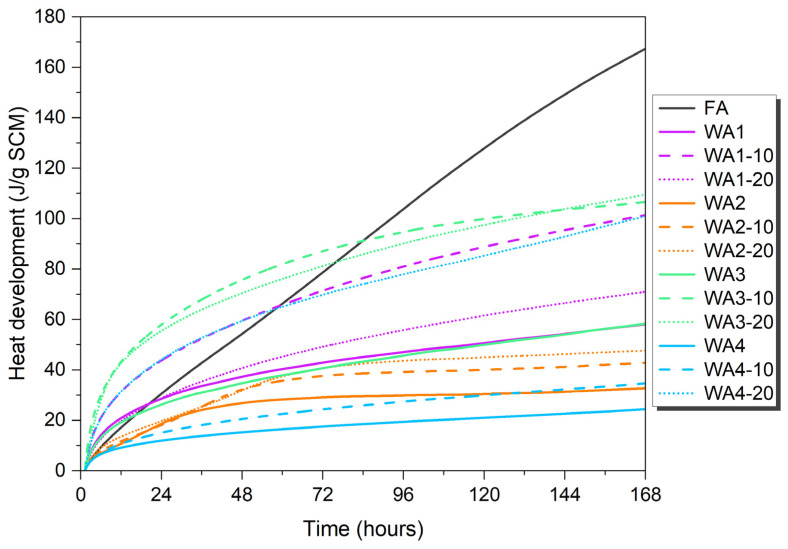
Cumulative heat release over 7 days at 40 °C for R3 paste samples containing unground and ground wood ashes (WA3 and WA4) for different grinding durations (10 and 20 min) and fly ash (FA).

The cumulative heat release of the wood ashes and fly ash is given in Figure 6. The highest value was recorded for FA (168.15 J/g), followed by WA3-20 (109.73 J/g), WA3-10 (106.73 J/g), WA1-10 (101.66 J/g), WA4-20 (101.56 J/g), WA1-20 (71.22 J/g), WA1 (58.31 J/g), WA3 (58.57 J/g), WA2-20 (47.75 J/g), WA2-10 (42.93 J/g), WA4-10 (34.79 J/g), WA2 (32.81 J/g), and WA4 (24.54 J/g) after 168 h.

Although ASTM C1897-20 [44] does not specify a pozzolanic reactivity threshold, Suraneni [72] suggested a benchmark of 100 J/g. Based on this criterion, only FA, WA3-20, WA3-10, WA1-10, and WA4-20 exceeded this threshold and showed reactivity according to the R3 test.

The cumulative heat release increased with longer grinding durations, except for WA1. WA1 ground for 10 min (WA1-10) released more heat than WA1 ground for 20 min (WA1-20). WA1-10 also exhibited a faster reaction according to the Frattini test.

Kalina et al. [73] also classified materials as inert if their heat release was less than 100 J/g SCM, moderately pozzolanic if between 100 J/g SCM and 200 J/g SCM, and highly pozzolanic if exceeding 200 J/g SCM. Interestingly, WA4-10 showed very low heat release despite its relatively high compressive strength. The grinding process might not only increase the reactivity but also increase the possibility of using it as a filler and creating nucleation sites with fine particles [12,61]. Fillers are not reactive and do not undergo any reaction themselves. Nonetheless, using wood ash as a filler can still positively impact its suitability in cement-based materials. An inert filler can enhance the properties of a cement-based material by filling the voids between the cement grains in the mixture. Moreover, it can also contribute by providing nucleation sites for the hydrates in cement, accelerating the hydration reaction and improving the compressive strength development. Moreover, such a filler can also promote non-uniform nucleation, serving as a site for the formation of C-S-H and other hydrates [61,65,74]. This accelerates the process of hydration and improves the development of compressive strength [27,61]. This might be an explanation for the higher compressive strength of WA4-10.

On the other hand, WA4-20 released unexpectedly high cumulative heat compared to WA4 and WA4-10, possibly due to the amorphization of calcite, as given in Figure 3b. Moreover, WA4 contained significant amounts of CaO (29 wt.%). However, the content of SiO_2_, which is important for pozzolanic reactions, was very low (0.75 wt.%). These findings also support the possible hydraulic properties of WA4. These observations support previous findings in the literature indicating that calcium-rich wood ashes can exhibit hydraulic properties [14,24,49,75].

**Figure 7:**
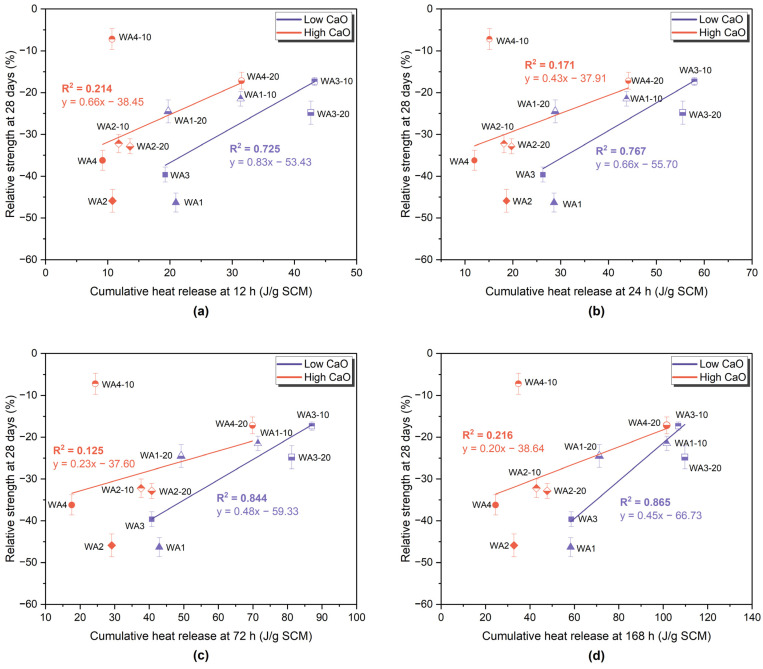
Correlation between the relative compressive strength at 28 days and cumulative heat release in the R^3^ test at (**a**) 12 h, (**b**) 24 h, (**c**) 72 h, and (**d**) 168 h. Wood ash-containing samples were grouped by CaO content into low-CaO (<20%) (WA1, WA1-10, WA1-20, WA3, WA3-10, WA3-20) and high-CaO (>20%) (WA2, WA2-10, WA2-20, WA4, WA4-10, WA4-20). Linear regression lines and corresponding R^2^ values are shown for each group.

Figure 7 shows the correlation between the relative compressive strength at 28 days and the cumulative heat release in the R3 test at 40 °C over four selected time intervals [76]: (a) 12 h, (b) 24 h, (c) 72 h, and (d) 168 h. The samples were categorized by their CaO content into low-CaO (<20%), which included WA1, WA1-10, WA1-20, WA3, WA3-10, and WA3-20, and high-CaO (>20%), which included WA2, WA2-10, WA2-20, WA4, WA4-10, and WA4-20. Linear regression lines and the corresponding R^2^ values are shown separately for each group.

For the low-CaO ashes, a strong correlation was observed between the cumulative heat and 28-day SAI, which increased over time, with R^2^ values of 0.725, 0.767, 0.844, and 0.865 at 12, 24, 72, and 168 h, respectively. These results indicate that the cumulative heat release in the R3 system reflects pozzolanic reactivity, which contributes to long-term strength. The strong correlation at 168 h indicates that pozzolanic reactions not only begin early but also continue and remain measurable well into later hydration stages. This trend aligns with the findings of Londono-Zuluaga et al. [77], who reported a strong relationship between heat release and the relative compressive strength across a wide range of SCMs, confirming the value of the R^3^ test parameters in predicting reactivity.

In the high-CaO group, low R^2^ values were consistently observed across all time intervals (≤0.22). This poor correlation is likely due to their low reactive silica content and the dominance of filler or hydraulic effects, which contribute to strength without relying significantly on pozzolanic reactivity. As highlighted by Suraneni [72], separating latent hydraulic and inert behavior in such systems requires additional measurements, such as CH consumption.

### 3.5. Phase Development

**Figure 8:**
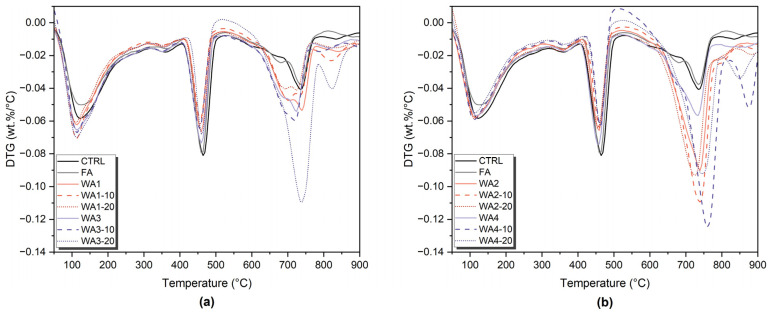
DTG curves of 28-day-old pastes containing unground and ground (**a**) WA1 and WA3 and (**b**) WA2 and WA4, compared to CTRL and FA-containing pastes.

**Figure 9:**
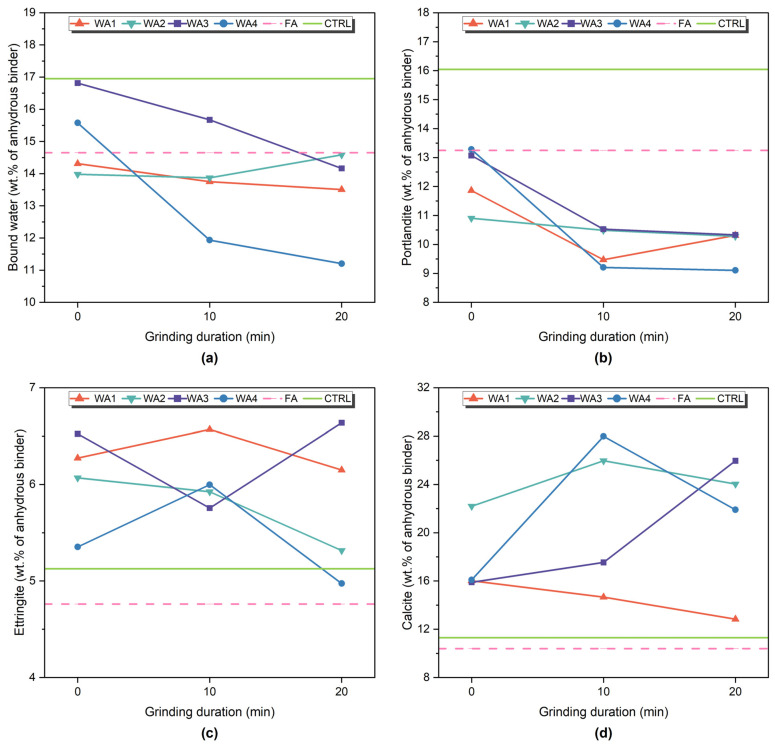
Quantification of (**a**) bound water, (**b**) portlandite, (**c**) ettringite (estimated), and (**d**) calcite content of 28-day-old paste samples based on TGA analysis.

**Figure 10:**
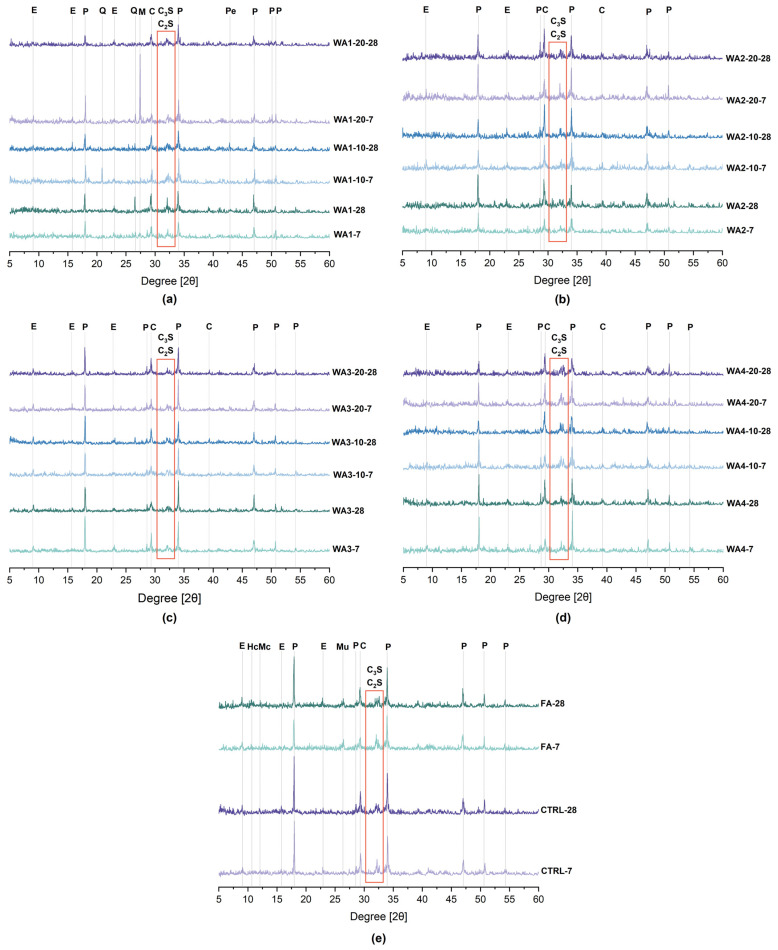
XRD patterns of 7- and 28-day-old pastes containing: (**a**) WA1, WA1-10, WA1-20; (**b**) WA2, WA2-10, WA2-20; (**c**) WA3, WA3-10, WA3-20; and (**d**) WA4, WA4-10, WA4-20, as well as (**e**) CTRL and FA-containing pastes. (P = Portlandite, C = Calcite, Q = Quartz, E = Ettringite, Pe = Periclase, M = Microcline, Mu = Mullite, C_3_S = Alite, C_2_S = Belite, Hc = Hemicarbonate, Mc = Monocarbonate). The orange box highlights the 2θ region corresponding to alite (C_3_S) and belite (C_2_S).

Phase development in the 28-day-old pastes was investigated using TGA/DTG and XRD analyses. The DTG curves are presented in Figure 8, showing thermal decomposition events within three main regions: 50–120 °C, attributed to the dehydration of ettringite and loosely bound C-S-H (and other phases that may decompose within this range); 400–550 °C, corresponding to the dehydroxylation of portlandite (CH); and 600–800 °C, associated with the decarbonation of calcite and other carbonate phases [46,78]. The quantified content of bound water, portlandite, ettringite, and calcite is given in Figure 9. In addition, the XRD patterns of the 7- and 28-day-old pastes are shown in Figure 10.

The control sample (CTRL), which contained only OPC, showed the highest level of hydration, with 16.95% bound water and 16.04% portlandite. These values reflect normal cement hydration without dilution or additional reactions. The XRD analysis confirmed the strong portlandite peaks and the presence of ettringite. These results served as benchmarks to evaluate the hydration and reactivity of fly ash- or wood ash-containing samples.

Since all ash-containing pastes were prepared by replacing 20 wt.% of cement, part of the reduction in portlandite content was due to the dilution effect. Lower CH content is expected because less cement is present in the mix, even without its reaction [27,48,78]. Portlandite consumption is one of the key indicators of pozzolanic reactions [8]. However, CH consumption alone is not sufficient to assess pozzolanic activity [79]. Other parameters, such as the bound water content, ettringite formation, heat release, compressive strength, and Frattini test results, must also be considered to better understand whether the changes are due to actual reactions or related to dilution or carbonation [46,78,79].

In the FA sample, portlandite content decreased to 13.68%, and the bound water was also lower (14.65%) than in the CTRL. This decrease can be explained by dilution and pozzolanic reactions, as fly ash contains amorphous aluminosilicates that react with CH to form additional C-S-H [27,79,80]. The ettringite (4.76%) and calcite (10.39%) content were slightly lower than in the CTRL. These trends are consistent with the low reactivity of low-Ca (Class F) fly ashes, which usually show slow pozzolanic reactions at early ages [8,80]. The XRD patterns showed decreased CH peak intensities, and ettringite was still present.

Among the wood ash-containing samples, WA3-10 showed the most apparent pozzolanic activity. The CH content decreased from 13.07% to 10.53%, while bound water remained relatively high at 15.67%. The ettringite content was 5.76%, and calcite slightly increased. These results suggest that WA3-10 reacted with CH to form additional C-S-H and possibly other hydrates. The CH peaks in XRD were weaker than those of the unground WA3 sample, and ettringite was still visible. WA3-10 also had the highest heat release (103.6 J/g SCM) and compressive strength among all wood ash-containing samples. This performance is also linked to its fine particle size (10.9 µm) and large surface area (1021 m^2^/kg), which enhance both reactivity and microstructural packing [46]. Its low D10 value (2.77 µm) also suggests a large amount of ultrafine particles, which may contribute to pozzolanic activity. On the other hand, WA3-20 consumed slightly more CH (10.18%) but showed less bound water (14.16%) and lower compressive strength, suggesting that longer grinding durations, despite the finer particle size, do not necessarily enhance the reactivity and may increase carbonation instead.

WA1 also showed moderate pozzolanic behavior, especially after grinding for 10 min. The CH content decreased from 11.86% to 9.49%, and ettringite increased to 6.57%. However, bound water slightly decreased from 14.31% to 13.75%, which might indicate that the strength improvements were also supported by physical effects such as particle packing or nucleation, rather than only by hydration [81]. The heat release (95.6 J/g SCM) and SAI results also comply with this conclusion. The XRD patterns showed lower CH peaks in the ground samples, whereas ettringite appeared unchanged.

On the other hand, WA2 showed minimal reactivity. After grinding, the CH content remained at around 10.4%, and bound water showed a small change. At the same time, the calcite content increased significantly, reaching 25.97% in WA2-10. The XRD results also supported this increase, where the calcite peaks became more prominent, while the CH peak intensity remained high. These results suggest that carbonation was the dominant process, rather than a pozzolanic or hydraulic reaction. Carbonation occurs when CH or Ca-rich hydrates react with atmospheric CO_2_ to form calcite, which consumes CH without contributing to the strength [78]. The poor reactivity of WA2 is supported by its low SiO_2_ (6.63%) and high CaO content (22%), combined with its coarse particle size and relatively small surface area. Its low SAI and limited heat release confirm this behavior. These observations align with earlier studies reporting that fineness alone does not ensure higher reactivity unless it is chemically supported by reactive components such as silica or alumina [27].

WA4 exhibited more complex behavior. The CH content decreased significantly with grinding (down to 8.87% in WA4-20), but bound water remained low (11.20%). The ettringite content stayed above 5%, and calcite increased significantly to 27.99% in WA4-10. These results suggest that WA4 underwent a combination of reactions: some CH may have been consumed by the early hydration of CaO, while calcite increased due to carbonation. The heat release of WA4-20 (93.2 J/g SCM) and the improved compressive strength also support the possible hydraulic activity. WA4 had the highest CaO content (29%) and a very high CaO/SiO_2_ ratio (38.67), which could explain its hydraulic nature. Sigvardsen et al. [49] stated that high-CaO ashes may behave as hydraulic binders or show limited pozzolanic activity, especially at early ages. The XRD analysis showed weakened portlandite peaks, a higher calcite peak intensity in WA4-10, and the presence of ettringite (6.00%), thus suggesting that reactive aluminates may have slowly contributed to early hydration and strength development [48].

Moreover, alite (C_3_S) and belite (C_2_S) were detected by XRD in all unground and ground WA2- and WA4-containing samples and, to a lesser extent, in some WA1 and WA3 samples. Their presence in the XRD patterns may indicate the limited hydration of clinker phases [46,78].

**Figure 11:**
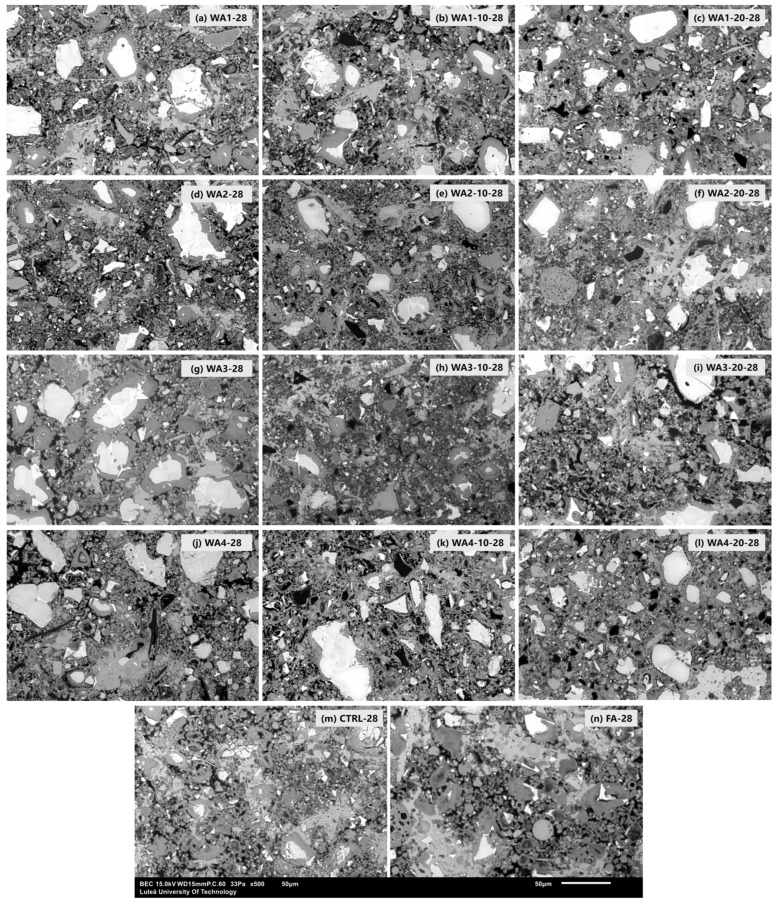
SEM images of 28-day-old paste samples containing 20 wt.% unground and ground wood wood ash, fly ash, or 100 wt.% cement at 500× magnification. The microstructure and distribution of unreacted particles varied depending on the type of wood ash and the grinding duration. Pastes containing ground wood ash exhibited a denser matrix and reduced porosity, indicating an enhanced microstructure.

The SEM analysis was conducted on resin-impregnated and polished paste samples. Figure 11 shows the backscattered electron (BSE) micrographs of 28-day-old pastes obtained at 500× magnification, illustrating the impacts of the wood ash type and grinding duration on the microstructure. Unreacted wood ash particles were seen in all samples. Among them, WA3-10-28, WA4-20-28, and WA1-20-28 displayed denser and more uniform matrices, with fewer visible voids and a more compact appearance. This suggests that finer particles and higher reactivity contributed to improved hydration and packing of the matrix. In contrast, pastes with unground wood ash showed more porous and heterogeneous structures and discontinuous hydration products, suggesting limited reactivity and slower microstructural development. These microstructures are related to the mechanical performance, as denser matrices and refined pore structures promote higher C-S-H formation and consequently higher compressive strength.

**Figure 12:**
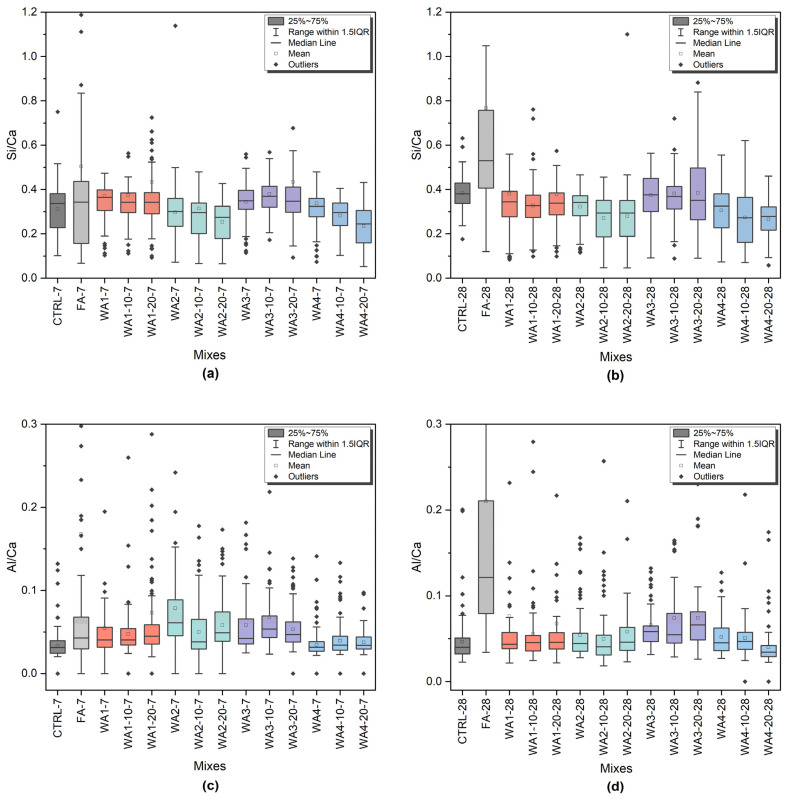
Elemental ratios (Si/Ca and Al/Ca) of C-S-H phases in SEM/EDS analysis of polished paste samples at 7 and 28 days. (**a**,**b**) Si/Ca ratios and (**c**,**d**) Al/Ca ratios for each mix at 7 days (left) and 28 days (right). Box colors indicate different mix types: dark grey represents the CTRL, light grey corresponds to FA-containing pastes, while orange, green, purple, and blue represent pastes containing WA1, WA2, WA3, and WA4, respectively, including both unground and ground forms.

To better understand the chemical structure of the hydration products, SEM/EDS analysis was carried out on polished paste samples. The focus was on the Si/Ca and Al/Ca ratios, which are useful indicators of the composition of the C-S-H gel and the extent of pozzolanic activity [52]. Figure 12 shows box plots of these ratios for all mixes at 7 and 28 days.

At 7 days, CTRL-7 had a narrow Si/Ca distribution, with a median around 0.35, and low Al/Ca values. This is typical for early-age Portland cement hydration. The FA-7 mix showed much broader ranges in both Si/Ca and Al/Ca, suggesting that pozzolanic reactions had already started and were contributing extra silica and alumina to the C-S-H gel.

Among the wood ash-containing samples, WA1-10 and WA3-10 showed higher values of Si/Ca and Al/Ca. Although not consistent across all data points, these higher values suggest that, in some areas, the wood ash had started to react and change the local gel structure, possibly forming C-A-S-H or AFt-like phases [46,52]. WA2-containing mixes showed a more moderate increase in the Si/Ca ratio with grinding, while WA4 mixes remained largely similar to CTRL, with low ratios and limited variability.

By 28 days, these differences became clearer. The FA-28 mix exhibited the widest spread and highest medians in both Si/Ca and Al/Ca, indicating ongoing pozzolanic reactions and continued gel development [46]. The WA3-20 and WA1-20 mixes also exhibited increased values and wider distributions, especially for Si/Ca. This suggests that grinding helped to activate their pozzolanic potential over time. WA2 still showed only limited changes, and WA4 remained almost unchanged, with values still close to those of CTRL.

The box plots confirm that mechanical grinding enhanced the reactivity of WA3 and slightly improved that of WA1. These mixes developed more reactive gel structures over time, with higher Si/Ca ratios and some localized Al incorporation. WA2 showed a moderate improvement, but WA4 remained mostly inert, likely acting as a physical filler rather than a reactive binder.

These findings highlight the importance of both the chemical composition and grinding in determining how effective wood ash can be as a supplementary cementitious material. WA3, which has higher silica content and responds well to grinding, seems to be the most promising candidate among the wood ashes studied.

## 4. Conclusions

This study investigated the pozzolanic properties of four different wood ashes (WA1, WA2, WA3, WA4), each ground for 10 and 20 min. A comparative analysis was also conducted with their unground forms and fly ash (FA) to evaluate changes in reactivity through various tests, including the SAI, Frattini test, R3 test, TGA/DTG, XRD, and SEM/EDS. The results showed that grinding significantly reduced the particle sizes of all wood ash samples. Grinding for 20 min did not increase the fineness of some samples, likely due to particle agglomeration. While none of the wood ashes met the pozzolanic oxide content requirement defined in EN 450-1 [58], some ground ashes exhibited pozzolanic activity based on the SAI, Frattini test, and R3 test results.

The strength activity index (SAI) results showed a significant improvement in compressive strength due to grinding. The statistical analysis also confirmed this: the one-way ANOVA revealed significant differences in compressive strength between the mixes at both 7 and 28 days (*p* < 0.0001), and the Tukey test showed that samples with ground wood ash had significantly higher strength than those with unground wood ash. WA3-10 achieved the highest 28-day compressive strength (43.3 MPa), which was 46.2% higher than that of unground WA3. Similarly, ground WA1 and WA4-containing mortars also exceeded the 75% SAI threshold, meeting the ASTM C618 [41] criteria for pozzolanic materials. All unground ashes and ground WA2-containing mortars remained below this threshold, suggesting either limited reactivity or the predominance of hydraulic or filler effects. These trends support previous findings in the literature indicating that grinding enhances pozzolanic properties through increased surface areas, filler packing, and amorphization.

The Frattini test results confirmed the pozzolanic behavior of unground and ground WA1, as well as ground WA3. Ground WA2 and WA4 showed some reactivity at later ages; however, their chemical compositions, characterized by low SiO_2_ and high CaO content, indicated hydraulic behavior rather than pozzolanic. Furthermore, portlandite consumption was observed in all wood ash samples, especially after grinding, as confirmed by the TGA and XRD analysis. These results demonstrate that both chemical and physical activation pathways contribute to the development of reactivity in wood ash systems.

The R3 test highlighted differences between pozzolanic and hydraulic contributions. WA3-10 and WA3-20 released 106.7 J/g and 109.7 J/g SCM, respectively. On the other hand, WA4-20 also exceeded this value (101.6 J/g SCM), but its low SiO_2_ content and high CaO indicated hydraulic reactivity. A strong correlation (R^2^ = 0.865) was observed between the 28-day compressive strength and the 168 h cumulative heat in low-CaO ashes (WA1, WA3). In contrast, this correlation was weak (R^2^ ≤ 0.22) for high-CaO ashes (WA2, WA4), emphasizing the different reactivity mechanisms.

The TGA/DTG and XRD analyses further confirmed these trends. The portlandite content in WA3-10 and WA1-10 decreased to 10.5% and 9.5%, respectively, compared to 16.0% in the control (CTRL), while bound water and ettringite formation increased. These results highlight adequate CH consumption and hydrate formation in ground low-CaO ashes, supporting pozzolanic reactivity. Although portlandite consumption was observed for WA2 and WA4, the high calcite formation in WA2 and WA4 suggests carbonation or hydraulic effects rather than pozzolanic activity.

The SEM/EDS analysis provided microstructural evidence of these differences. Ground WA1 and WA3 pastes exhibited denser matrices, fewer voids, and broader Si/Ca and Al/Ca elemental distributions, which are consistent with C-S-H and C-A-S-H formation.

Overall, the findings highlight the potential of 10 min of grinding to significantly enhance the reactivity of chemically suitable wood ashes like WA1 and WA3, while also emphasizing the limitations posed by high-CaO, low-SiO_2_ ashes such as WA2 and WA4. The combination of various test methods offers a comprehensive understanding of the activation mechanisms involved.

This study represents an initial investigation into the influence of the grinding process on the reactivity of wood ash. The findings show that, depending on its chemical composition, wood ash can be effectively utilized in cement-based materials by enhancing its pozzolanic or hydraulic reactivity through grinding.

While the results are promising and diverse, a limitation of this study is that it focused on four types of wood ash from only two sources; thus, it may not fully represent the broader variability observed in wood ashes from different origins and combustion processes. To build on the current findings, future research is recommended to include a wider range of wood ashes with diverse characteristics. It would also be valuable to investigate a broader set of grinding parameters. The effects of ground wood ash on the long-term durability of concrete should be further examined. Additionally, assessing the environmental impacts, such as energy consumption and emissions associated with grinding, could provide a more comprehensive understanding of the material’s suitability for sustainable construction.

## Figures and Tables

**Table 1:** Production parameters of wood ash samples WA1, WA2, WA3, and WA4, including biofuel composition, combustion technology, and combustion temperature. WA1, WA2, and WA3 originated from the same combustion facility but were collected during different operational periods, while WA4 was obtained from a different source.

Wood Ash	Biofuel Composition	Combustion Technology	Combustion Temperature (°C)
WA1	60% bark, 30% sawdust, and 10% dry wood chips	Grate combustion	1050
WA2	60% bark, 30% sawdust, and 10% dry wood chips	Grate combustion	1050
WA3	60% bark, 30% sawdust, and 10% dry wood chips	Grate combustion	1050
WA4	30% bark and 70% wood shavings	Grate combustion	800–900

**Table 2:** Chemical composition of wood ashes (WA1, WA2, WA3, WA4), fly ash (FA), and Portland cement (CEM I).

Chemical Composition (%)	WA1	WA2	WA3	WA4	FA	CEM I
SiO_2_	31.7	6.63	22.4	0.75	60.20	21.20
Al_2_O_3_	7.25	3.48	6.75	0.37	17.70	3.40
Fe_2_O_3_	4.19	1.11	2.62	0.17	2.78	4.10
CaO	14.7	22	15.1	29	1.37	63.30
K_2_O	5.14	9.10	8.25	1.20	1.61	0.56
MgO	2.08	3.43	2.69	2.52	0.43	2.20
MnO	0.61	1.22	0.80	1.80	0.05	-
P_2_O_5_	1.39	2.63	2.81	2.11	0.07	-
TiO_2_	0.33	0.12	0.3	0.01	0.71	-
Na_2_O	1.53	0.37	1.46	0.03	0.60	0.18
SO_3_	-	-	-	-	-	2.70
LOI (1000 °C)	34.5	43.7	29.7	56.7	1.80	2.50
SiO_2_ + Al_2_O_3_ + Fe_2_O_3_	43.14	11.22	31.77	1.29	80.68	28.70
CaO/SiO_2_	0.46	3.32	0.67	38.67	0.02	2.98
(CaO + MgO)/SiO_2_	0.53	3.84	0.79	42.03	0.02	3.08

**Table 3:** Equations and parameters used to quantify bound water, portlandite, ettringite, and calcite content from TGA measurements.

Phase	Temperature Range (°C)	Measured (wt.%)	Anhydrous (wt.% of Anhydrous Binder)
Bound Water	50–550	$H_{measured}={WL}_{50-550}$	$H_{anhydrous}=\frac{H_{measured}}{Weight\;at\;550\;{}^{\circ}C\;}\times 100$
Portlandite (Ca(OH)_2_)	400–550	${CH}_{measured}={WL}_{400-550}\times \frac{74}{18\;}$	${CH}_{anhydrous}=\frac{{CH}_{measured}}{Weight\;at\;550\;{}^{\circ}C\;}\times 100$
Ettringite (C_6_As_3_H_32_) (estimated)	50–120	${Ett}_{measured}={WL}_{50-120}\times \frac{1255}{32\;\times \;18}$	${Ett}_{anhydrous}=\frac{{Ett}_{measured}}{Weight\;at\;550\;{}^{\circ}C\;}\times 100$
Calcite (CaCO_3_)	600–800	${Cc}_{measured}={WL}_{600-800}\times \frac{100}{44\;}$	${Cc}_{anhydrous}=\frac{{Cc}_{measured}}{Weight\;at\;550\;{}^{\circ}C\;}\times 100$

Molar masses (g/mol): Ca(OH)_2_—74, H_2_O—18, C_6_As_3_H_32_—1255, CaCO_3_—100, CO_2_—44.

**Table 4:** Overview of test methods, sample compositions, and measurement descriptions.

Test	Sample Type	Mix Design	Description
SAI [41]	Mortar	0 wt.%, 20 wt.% WA/FA w/b ≈ 0.49 (0 wt.); 0.84 (20 wt.%)	Compressive strength of 50 mm cubes at 7 and 28 days
Frattini Test [42]	Paste	0 wt.%, 20 wt.% WA/FA	Cement + SCM + distilled water; 100 mL, sealed at 40 °C; filtrate titrated at 8, 15, 28 days
R3 Test [44]	R3 Paste	100 wt.% WA/FA	SCM + Ca(OH)_2_ + CaCO_3_ (1:3:0.5) in K^+^ solution; L/S = 1.2; sealed ampoules, 40 °C for 7 days
TGA	Paste	0 wt.%, 20 wt.% WA/FA w/b = 0.4	28-day hardened pastes; solvent-exchanged and analyzed between 20 and 1000 °C under Ar
XRD	Powder	WAs, FA, CEM I	Raw materials analyzed to identify original crystalline phases
	Paste	0 wt.%, 20 wt.% WA/FA w/b = 0.4	7- and 28-day hardened pastes analyzed for hydration products
SEM/EDS	Powder	WAs	Wood ash powders on conductive tape, morphology analysis
	Paste	0 wt.%, 20 wt.% WA/FA w/b = 0.4	7- and 28-day hardened pastes, BSE imaging and EDS analysis of C-S-H

**Table 5:** Particle size distribution parameters (D10, D50, D90) and specific surface areas (SSAs) of unground and ground wood ashes (WA1, WA2, WA3, WA4), fly ash (FA), and Portland cement (CEM I).

Material	Grinding Duration (min)	D10 (µm)	D50 (µm)	D90 (µm)	SSA (m^2^/kg)
WA1	0	15.7	97.2	581	172.4
WA1-10	10	6.91	29.5	159	444.3
WA1-20	20	5.49	20.6	125	566.5
WA2	0	15.6	103	629	186.6
WA2-10	10	3.53	22.2	147	829.6
WA2-20	20	6.03	22.8	150	519.3
WA3	0	18.7	102	581	143
WA3-10	10	2.77	10.9	36.3	1021
WA3-20	20	2.98	8.98	23.5	1048
WA4	0	18.8	139	532	131.9
WA4-10	10	4.16	12.7	39.5	782.1
WA4-20	20	3.78	11	31.1	882.9
FA	0	4.09	25.6	111	666.6
CEM I	0	4.34	22.4	80.3	672.9

**Table 6:** Frattini test results with [CaO] reduction after 8, 15, and 28 days.

Sample	Days	[OH] (mmol/L)	[CaO] (mmol/L)	Theoretical Max. [CaO] (mmol/L)	[CaO] Reduction (%)
CTRL	8	50.1	10.0	10.0	0 (−0.5)
FA	8	47.5	8.0	10.8	25.7
WA1	8	66.2	9.8	6.8	0 (−43.2)
	15	56.4	7.0	8.5	17.8
WA1-10	8	54.9	6.7	8.8	23.3
WA1-20	8	63.3	8.9	7.2	0 (−23.1)
	15	69.8	5.9	6.4	8.0
WA2	8	108.5	7.6	3.7	0 (−103.1)
	15	94.4	6.8	4.4	0 (−54.6)
	28	87.5	5.7	4.8	0 (−18.1)
WA2-10	8	102.4	5.5	4.0	0 (−38.0)
	15	85.8	5.3	4.9	0 (−7.9)
	28	75.6	5.1	5.8	10.5
WA2-20	8	104.3	5.8	3.9	0 (−48.9)
	15	87.5	5.1	5.8	0 (−6.2)
	28	73.0	4.8	6.0	19.3
WA3	8	90.7	5.5	4.6	0 (−17.9)
	15	85.8	6.9	4.9	0 (−39.2)
	28	78.4	7.8	5.5	0 (−40.7)
WA3-10	8	91.2	3.4	4.6	26.3
WA3-20	8	86.4	6.7	4.9	0 (−36.9)
	15	83.9	4.3	5.1	16.3
WA4	8	57.6	12.5	8.2	0 (−51.8)
	15	60.0	10.7	7.8	0 (−37.6)
	28	64.0	8.7	7.1	0 (−22.0)
WA4-10	8	56.0	10.0	8.5	0 (−17.8)
	15	55.7	9.9	8.6	0 (−14.6)
	28	55.5	8.0	8.6	7.8
WA4-20	8	64.6	11.5	7.1	0 (−63.2)
	15	56.5	10.2	8.4	0 (−21.4)
	28	64.7	5.8	7.0	17.0

## Data Availability

The original contributions presented in this study are included in the article. Further inquiries can be directed to the corresponding author.
